# Supplementary material for: Dynamics of apomictic and sexual reproduction during primary succession on a glacier forefield in the Swiss Alps
Source: Sci Rep. 2020 May 19;10:8269. doi: 10.1038/s41598-020-64367-9 (PMC7237654; doi:10.1038/s41598-020-64367-9)
Supplement: Supplementary file 6 — Supplementary Figure 1. [file 41598_2020_64367_MOESM6_ESM.pdf]

# Dynamics of apomictic and sexual reproduction during primary succession on a glacier forefield in the Swiss Alps

**Christian Sailer[1,3], Jürg Stöcklin[2], and Ueli Grossniklaus[1]***

[1] Department of Plant and Microbial Biology & Zurich-Basel Plant Science Centre, University of Zurich, Zollikerstrasse 107, 8008 Zurich, Switzerland
[2] Institute of Botany & Zurich-Basel Plant Science Centre, University of Basel, Schönbeinstrasse 6, 4056 Basel, Switzerland
[3] Current address: Institute of Integrative Biology, ETH Zurich, Zurich, Switzerland

* grossnik@botinst.uzh.ch, Tel. +4463 48240

## Supplementary Figures

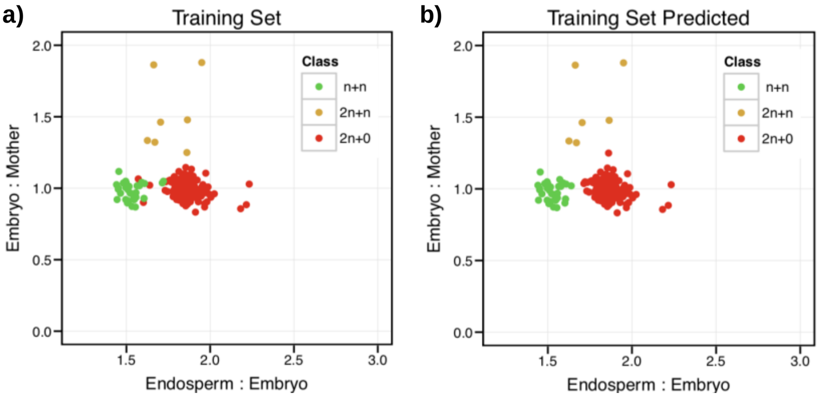


c)

| | n+n | 2n+n | 2n+0 |
|---|---|---|---|
| n+n | 188 | 0 | 12 |
| 2n+n | 0 | 70 | 0 |
| 2n+0 | 4 | 0 | 196 |

**Supplementary Figure 1.** Linear discriminant analysis to distinguish the developmental pathway via which a seed was produced. **a)** Training set used for linear discriminant analysis. The offspring classes were assigned manually based on the flow cytometric histograms. **b)** Linear discriminant analysis of the training set using the identified parameters. **c)** Number of correctly and wrongly assigned offspring.

## Data generated and analysed

**Dataset_Morteratsch** Dataset containing the phenotyping information of all sampled individuals.

**Dataset_GT_LOA267** Genotype matrix of marker LOA267. Column names are binned fragment sizes.

**Dataset_GT_SSR3** Genotype matrix of marker SSR3. Column names are binned fragment sizes.

**Dataset_GT_SSR42** Genotype matrix of marker SSR42. Column names are binned fragment sizes.

**Dataset_GT_SSR87** Genotype matrix of marker SSR87. Column names are binned fragment sizes.
